# Development of UHPLC-MS/MS Method for Indirubin-3′-Oxime Derivative as a Novel FLT3 Inhibitor and Pharmacokinetic Study in Rats

**DOI:** 10.3390/molecules25092039

**Published:** 2020-04-27

**Authors:** Na Yoon Kim, Yong-Chul Kim, Yoon Gyoon Kim

**Affiliations:** 1 College of Pharmacy, Dankook University, Cheonan 31116, Korea; hjly226@daum.net; 2 Gwangju Institute of Science and Technology (GIST), School of Life Sciences, Gwangju 61005, Korea; yongchul@gist.ac.kr

**Keywords:** LDD-2614, novel FLT3 inhibitor, indirubin derivative, LC-MS/MS, pharmacokinetics

## Abstract

This study aimed to develop and validate a sensitive liquid chromatography-coupled tandem mass spectrometry method for the quantification of LDD-2614, an indirubin derivative and novel FLT3 inhibitor, in rat plasma. In addition, the developed analytical method was applied to observe the pharmacokinetic properties of LDD-2614. Chromatographic separation was achieved on a Luna omega C_18_ column using a mixture of water and acetonitrile, both containing 0.1% formic acid. Quantitation was performed using positive electrospray ionization in a multiple reaction monitoring (MRM) mode. The MRM transitions were optimized as *m/z* 426.2→113.1 for LDD-2614 and *m/z* 390.2→113.1 for LDD-2633 (internal standard), and the lower limit of quantification (LLOQ) for LDD-2614 was determined as 0.1 ng/mL. Including the LLOQ, the nine-point calibration curve was linear with a correlation coefficient greater than 0.9991. Inter- and intraday accuracies (RE) ranged from −3.19% to 8.72%, and the precision was within 9.02%. All validation results (accuracy, precision, matrix effect, recovery, stability, and dilution integrity) met the acceptance criteria of the U.S. Food and Drug Administration and the Korea Ministry of Food and Drug Safety guidelines. The proposed method was validated and demonstrated to be suitable for the quantification of LDD-2614 for pharmacokinetics studies.

## 1. Introduction

In acute myeloid leukemia (AML), the most common type of leukemia, leukocytes in the immature stage of bone marrow transform into malignant cells before becoming overgrown and spreading throughout the body through the blood. AML usually occurs in elderly patients [1,2]. The FMS-like receptor tyrosine kinase 3 (FLT3), a member of the receptor tyrosine kinase family, is an important target in the field of AML drug development [3]. FLT3 activates the intracellular tyrosine kinase domain, which dimerizes and autophosphorylates upon binding of the FLT3 ligand and causes phosphorylation of downstream molecules [4]. It also activates a variety of signaling pathways, including proliferation, survival, and differentiation of hematopoietic precursor cells [5]. FLT3 is mostly expressed in AML cells, where mutations occur in approximately 30% of cells [6]. Mutations that include internal tandem duplication occur in approximately 25% of AML cells, and point mutations in the tyrosine kinase domain occur in approximately 5% [6]. These mutations can cause chemotherapy failure and recurrence, and thus have a poor prognosis [7]. Studies have shown that the FLT3 internal tandem duplication mutation induces the progression of AML, making it an effective therapeutic target for the disease [8].

The many FLT3 inhibitors that have been studied can be classified into first- and second-generation inhibitors based on their specificity. First-generation inhibitors such as sunitinib [9], sorafenib [10], midostaurin [11], lestaurtinib [12], and tandutinib [13] exhibit no specificity toward FLT3. The inhibition of multiple receptor tyrosine kinases can enhance efficacy by inhibiting downstream or parallel signal transduction pathways of FLT3 or other targets of AML cells. However, multiple targeting and non-selective tyrosine kinase inhibitory actions can cause side effects [14,15]. Second-generation inhibitors, which include quizartinib [16], crenolanib [17], and gilteritinib [18] are more specific, potent, and less toxic in terms of non-selective effects. However, second-generation FLT3 inhibitors mainly target only FLT3 and have no effect on FLT3 downstream targets or parallel signal pathways in AML cells [19]. Therefore, there is a need for the continued development of potent and selective FLT3 inhibitors, particularly those that are effective against resistant AML caused by mutations. A previously reported joint research effort showed that indirubin analogs potently inhibited FLT3 [20]. Further optimizations were subsequently performed to yield a potent inhibitory activity against FLT3 [1,21]. On the basis of previously reported 3′-oxime moieties, it has been found that the 5,5′-substituted indirubin analogue LDD-2614 exhibited more optimized physicochemical properties to maintain FLT3 inhibitory activity.

In previous studies involving in vitro assays, LDD-2614 exhibited FLT3 inhibition, anti-proliferative activity in the AML line of MV4-11 cells, and significant in vivo antitumor activity in a MV4-11 xenograft animal model [22]. To assess the pharmacokinetic parameters of LDD-2614, including its oral bioavailability, proven bioanalytical methods are needed; however, no assays for the quantification of LDD-2614 have yet to be reported. In this paper, we report the pharmacokinetic properties of LDD-2614 after oral and intravenous administration in rats and detail the development and validation of a novel liquid chromatography-coupled tandem mass spectrometry (LC-MS/MS) method for the quantification of LDD-2614.

## 2. Results and Discussion

### 2.1. LC-MS/MS Optimization

**Figure 1:**
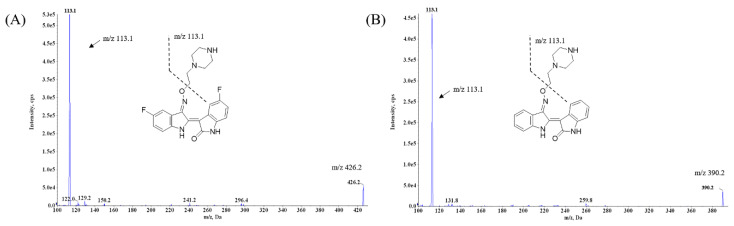
Structure and Q1 full scan product ion mass spectra of (**A**) LDD-2614 and (**B**) LDD-2633 (IS).

To obtain optimum mass spectrometer conditions, LDD-2614 and LDD-2633 (IS) solutions were directly injected into the mass spectrometer, and full scan mass spectra were collected in positive and negative ion modes. The full scan mass spectra showed that the ionization of LDD-2614 and LDD-2633 was more sensitive in the positive ion mode than in the negative ion mode. Figure 1 shows the mass spectra and chemical structures of LDD-2614 and LDD-2633. LDD-2633 was selected as an IS because it is an indirubin-type compound similar to LDD-2614 and is a drug that can be stably measured at high concentrations under positive ion mode and LDD-2614 separation conditions. LDD-2633 also showed no special interference effect with LDD-2614 and exhibited a stable extraction recovery. The optimized mass spectra of LDD-2614 and LDD-2633 showed intense [M + H]^+^ at *m/z* 426.2 and 390.2, respectively. These precursor ions were obtained as the most abundant and stable product ions through the optimization of energy parameters including declustering potential, collision energy, collision cell exit potential, and entrance potential. The optimized analytical parameters, including the mass transitions for LDD-2614 and LDD-2633, are summarized in Table 1. The product ion of LDD-2614, *m/z* 113.1, was expected to be a cleaved 1-ethylpiperazine fragment, as shown in Figure 1A. The *m/z* 113.1 product ion of the IS, which was chosen as the most sensitive, was also expected to be generated in the same reaction as LDD-2614 (Figure 1B).

Various types of mobile phase systems were tested to achieve the optimal response. First, we tried with methanol and acetonitrile to select organic solvents. The sensitivity was similar in both solvents, but acetonitrile resulted in a more reduced baseline than methanol. Then, formic acid and ammonium acetate buffers were used to identify peak shape improvements. The mobile phase consisting of acetonitrile and 10 mM ammonium acetate in water did not improve the peak shape. Thus, we chose acetonitrile in 0.1% formic acid and water in 0.1% formic acid as the mobile phase. The mobile phase was performed isocratic elution. After several tests with varying ratios, we found that a high ratio of acetonitrile improved the analyte response and peak shape. For chromatographic separation, various reversed phase columns including Gemini NX C_18_ (100 mm × 2.0 mm, 3 µm), Unison UK C_8_ (2.0 mm × 75 mm, 3 µm), Venusil XBP C_18_ (100 mm × 2.1 mm, 3 µm), Cadenza CD C_18_ (50 mm × 2.0 mm, 3 µm), and Luna Omega PS C_18_ (100 mm × 2.1 mm, 3 µm) columns were investigated. The Venusil XBP C_18_ and Luna Omega PS C_18_ columns were further considered, and the latter was ultimately chosen because it offered the highest intensity and better peak shapes and areas.

### 2.2. Sample Preparation

Various extraction procedures, including protein precipitation with acetonitrile and methanol and liquid-liquid extraction with ethyl acetate and methyl tert-butyl ether have been tested to remove interferences and to achieve a suitable analyte recovery for accurate concentration measurements of LDD-2614 in plasma. Protein precipitation with acetonitrile, which is widely used for the preparation of biological samples, also has shown good sensitivity. However, the sensitivity of the analyte was about 2.4 times higher in the liquid-liquid extraction with ethyl acetate than in protein precipitation extraction samples. In addition, thin peaks were observed. The recovery was also better for the ethyl acetate extraction (97.8–98.6%) than for the precipitation (43.3–46.6%) with acetonitrile sample. Therefore, in this study, a simple liquid-liquid extraction method was used. The use of ethyl acetate has been shown to be efficient for sensitive LDD-2614 extraction with adequate reproducibility and recovery.

### 2.3. Method Validation

#### 2.3.1. Specificity

**Figure 2:**
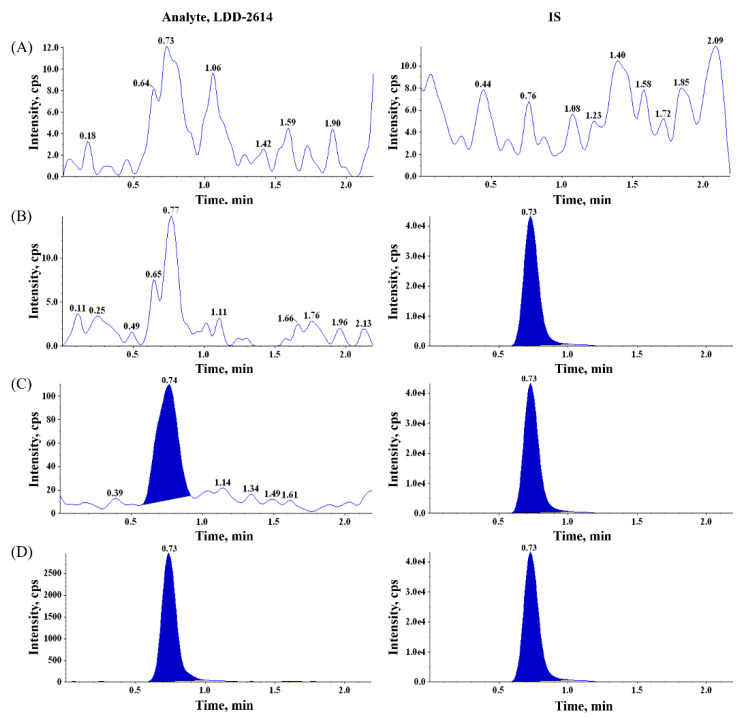
LC-MS/MS chromatograms of (**A**) double blank rat plasma; (**B**) blank rat plasma samples spiked with IS; (**C**) spiked at lower limit of quantification (LLOQ) level in rat plasma, 0.1 ng/mL; and (**D**) plasma sample obtained 480 min after oral administration of 1 mg/kg to rat.

The representative chromatograms of blank rat plasma, blank rat plasma spiked with the IS, blank rat plasma spiked with LDD-2614 at the lower limit of quantification (LLOQ) concentration, and real biosamples are shown in Figure 2. In the blank plasma, peaks which appeared to be endogenous components of the biological matrix appeared near the retention time of LDD-2614 but did not exceed 20% at the LLOQ concentration. The retention times for LDD-2614 and the IS were approximately 0.73 min. No interference or extraneous peaks were observed at the retention times of LDD-2614 and IS. The peak area of the IS was at least 20 times that of the blank signal at the same retention time as that of the IS.

#### 2.3.2. Matrix-Matched Calibration Curve and LLOQ

The matrix-matched calibration curve was evaluated by testing nine concentration levels on five consecutive days. The ratio of the peak areas of LDD-2614 and the IS was plotted to construct a curve by weighted 1/*x*^2^ regression and to determine the regression parameters. Typical equations describing each matrix-matched calibration curve are shown in Table 2. The matrix-matched calibration curve showed good linearity (*r*^2^ > 0.9991) in the concentration range of 0.1–500 ng/mL with precision (RSD) and accuracy (RE) values of 1.0–5.5% and −4.1–1.5%, respectively. The LLOQ of LDD-2614, defined as a signal-to-noise ratio of >5, was 0.1 ng/mL. The precision (RSD) of the LDD-2614 LLOQ was 5.5%, and the accuracy (RE) was 1.5%, indicating excellent sensitivity (Table 3).

#### 2.3.3. Precision and Accuracy

Testing of the QC sample of LDD-2614 was repeated five times at four different concentrations in plasma (0.1, 0.3, 150, and 400 ng/mL) to determine the intra- and interday accuracy and precision. The resulting data are summarized in Table 4. The intra- and interday accuracy (RE) was in the range of −3.2–8.7%, and the precision (RSD) was in the range of 3.0–9.0%. These results meet the biological analysis requirements of being within ±15% for all QC samples and within ±20% for the LLOQ.

#### 2.3.4. Recovery and Matrix Effect

Recovery was evaluated by comparing the QC samples prepared according to the sample pretreatment method and plasma samples spiked at the same concentration after extraction. The QC sample tests were replicated five times at different levels (LQC, MQC, and HQC). The extraction recovery in rat plasma was 103.9% for the LQC, 100.6% for the MQC, and 98.9% for the HQC. For the IS, the mean extraction recovery was 98.0%. The matrix effect was assessed by comparison with a standard solution of the same concentration as the spiked plasma sample after extraction. The matrix effects were 100.6%, 101.0%, and 99.5% for LDD-2614 and 98.1% for the IS. The recovery and matrix effects are summarized in Table 5. The results showed that there was no significant variation in recovery efficiency, and the matrix effect was negligible even though the retention of analytes in the column was short.

#### 2.3.5. Stability

Stability studies using three QC samples for freeze-thaw stability (three freeze-thaw cycles lasting 12 h each), long-term stability (sample stored at −80 °C and analyzed after 2 weeks), short-term temperature stability (samples kept at room temperature for 6 h), and post-preparation stability (12 h in autosampler) were evaluated. The measurements of the analytes were compared with those from newly prepared QC samples at the same concentrations. The stability results are summarized in Table 6. The accuracy (RE) of all analytes ranged from −0.6% to 8.6% for the three freeze-thaw cycles, −5.5% to −2.6% for long-term stability, −3.6% to −0.1% for short-term stability, and −1.5% to 1.5% for post-preparation stability. There was no significant degradation observed under the different conditions, indicating that LDD-2614 is stable during storage and analysis.

#### 2.3.6. Dilution Integrity

The accuracies of plasma samples diluted 50-fold and 100-fold with blank rat plasma were 90.9% and 91.8%, respectively, and the precision values were 4.1% and 4.8%. This demonstrates the ability to dilute samples up to 100-fold using this method.

### 2.4. Pharmacokinetic Studies

The validated LC-MS/MS method was successfully implemented to quantify LDD-2614 in Sprague Dawley rat plasma to perform a pharmacokinetic study on LDD-2614 in rats after oral and intravenous administration.

#### 2.4.1. Intravenous Study

**Figure 3:**
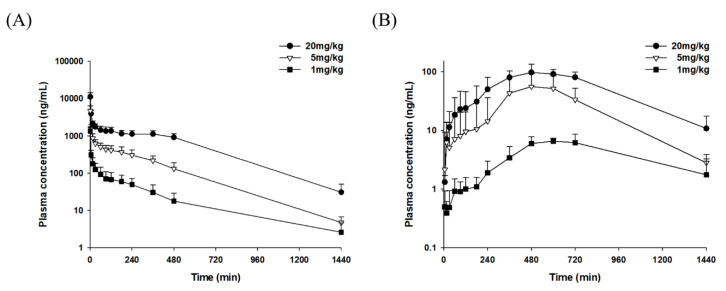
The mean plasma concentration-time profiles of LDD-2614 after (**A**) intravenous and (**B**) oral administration to rats at dose of 1 mg/kg (■, *n* = 7 and 6 for intravenous and oral administration, respectively); 5 mg/kg (▽, *n* = 5 and 6 for intravenous and oral administration); 20 mg/kg (●, *n* = 6 and 5 for intravenous and oral administration, respectively). Vertical bars represent standard deviation.

The plasma concentration-time profiles of LDD-2614 after intravenous administration to rats are shown in Figure 3, and the main pharmacokinetic parameters are displayed in Table 7. When LDD-2614 was administered intravenously, the terminal phase occurred approximately 3 h after administration in the dose range of 1–20 mg/kg. At this time, the half-life (T_1/2_) was approximately 3–4 h. The AUC_last_ calculated using the plasma concentration obtained after intravenous administration increased proportionally to the dose. There was no significant difference in the dose range of 1–20 mg/kg when AUC was normalized to the dose. In other words, linear pharmacokinetic characteristics were observed in the dose range used in this study.

#### 2.4.2. Oral Study

The mean plasma concentration-time profiles after oral administration are illustrated in Figure 3, and the main pharmacokinetic parameters are summarized in Table 7. Oral absorption of LDD-2614 showed erratic patterns. The plasma concentrations in all individual rats appeared to increase very slowly after oral administration. However, the plasma concentration increased sharply after 4–6 h and reached the maximum at 6–10 h. The AUC_last_ obtained from plasma concentrations after oral administration increased with dose but not proportionally. In particular, for the oral administration of 5 and 20 mg/kg, the AUC_last_ was 23.4 and 61.1 μg·min/mL, respectively, with a smaller increase (approximately 2.5-fold) in AUC as compared with the increase in dose (four-fold). There could be a variety of reasons for this unusual absorption pattern. One likely reason is that limited absorption windows can exist in the latter part of the gastrointestinal tract such as the jejunum and ileum rather than in the stomach or duodenum. Compared with the AUC_last_ obtained after intravenous administration, the bioavailability after oral administration was 7.1–11.7% at the doses used in this study.

## 3. Materials and Methods

### 3.1. Chemicals and Reagents

The LDD-2614 and LDD-2633 (used as an internal standard, IS) compound were synthesized at the Gwangju Institute of Science and Technology (Gwangju, Korea). HPLC grade acetonitrile and water were purchased from Honeywell Burdick and Jackson (Muskegon, MI, USA), and ethyl acetate was supplied from J.T. Baker (Avantor Performance Materials, Center Valley, PA, USA). Analytical reagent grade dimethyl sulfoxide (DMSO, purity ≥98%) and formic acid (purity ≥98%) were purchased from Sigma-Aldrich (St. Louis, MO, USA). Distilled water was purified with a Millipore Milli-Q system (Bedford, MA, USA). All other chemicals and reagents were of analytical grade.

### 3.2. Animals

Male Sprague Dawley rats (7–8 weeks, 230–250 g) were supplied from Samtaco (Osan, Korea) and were housed in cages before the experiments. All animal experiments were approved by the Dankook University’s Institutional Animal Care and Use Committee (approval no. DKU-19-029). All rats were kept in a light-controlled room maintained at a temperature of 23 ± 2 °C with 12 h light and dark cycles and a relative humidity of 50% ± 10%. The rats were given ad libitum access to standard food and water and acclimated to the environment for 7 days prior to the experiment.

### 3.3. Stock and Standard Solutions and Quality Control Samples

A stock solution of LDD-2614 was prepared in DMSO at a concentration of 1.0 mg/mL. The stock solution was serially diluted in 50% acetonitrile to prepare working solutions at the final concentrations of 1, 5, 10, 50, 100, 500, 1000, 2000, and 5000 ng/mL. Quality control (QC) samples were prepared in the same way to obtain final concentrations of 1 (lower limit of quantification QC, LLOQ), 3 (low QC, LQC), 1500 (mid QC, MQC), and 4000 ng/mL (high QC, HQC). The LDD-2633 (IS) solution was prepared in acetonitrile to a final concentration of 1 μg/mL. All solutions were stored at −20 °C until analysis.

### 3.4. Preparation of Calibration and QC Samples and Sample Preparation

Calibration samples were prepared by adding 5 μL of the working solutions to 45 μL of blank plasma to obtain the final concentrations of 0.1, 0.5, 1, 5, 10, 50, 100, 200, and 500 ng/mL. The QC samples were prepared in the same manner to the final concentrations of 0.1, 0.3, 150, and 400 ng/mL.

A liquid-liquid extraction method with ethyl acetate was used to extract LDD-2614 from the rat plasma. The IS solution (5 μL, 1 μg/mL) and 500 μL of ethyl acetate were added to 50 μL of the calibration samples and QC samples. The mixture was stirred for 1 min and centrifuged for 5 min at 12,000 rpm, under refrigerated conditions (at 4 °C). The upper layer was transferred to another microtube and evaporated under nitrogen gas at 25 °C in a MG 2100 Eyela dry thermo bath (Rikakikai Company, Tokyo, Japan). Finally, the residue was reconstituted in 100 μL of 0.1% formic acid in 90% acetonitrile, and 5 μL of the solution was injected into the LC-MS/MS system.

### 3.5. Instrumentation and LC-MS/MS Conditions

A Dionex Ultimate 3000 HPLC unit chromatography system (Thermo Fisher Scientific, Boston, MA, USA) coupled with an AB SCIEX API 3200 triple quadrupole mass spectrometer (Applied Biosystems Sciex, Toronto, Ontario, Canada) was used for the LDD-2614 analysis. Chromatographic separation was achieved using a Luna Omega PS C_18_ column (100 mm × 2.1 mm, 3 µm, Phenomenex, Torrance, CA, USA) and a Security Guard C_18_ cartridge (4 mm × 2.0 mm i.d., Phenomenex); the column oven temperature was set to 40 °C. The mobile phase consisting of 0.1% formic acid in water (phase A, 10%) and 0.1% formic acid in acetonitrile (phase B, 90%) was maintained isocratically at a flow rate of 0.25 mL/min. The injection volume was 5 μL, and the total analysis run time was 2.5 min for each sample.

The mass spectrometer was operated in a multiple reaction monitoring (MRM) mode with positive electrospray ionization. A standard solution of LDD-2614 (50 ng/mL) was infused directly into the mass spectrometer to optimize the source and compound parameter settings. The optimized MS/MS parameters were as follows: ion spray voltage, 5500 V; ion source gas 1 and 2, 50 psi; ion source temperature, 350 °C; curtain gas, 20 psi; collision gas, 5 psi. System control and data analysis were carried out with the Analyst software (Analyst 1.5.2).

### 3.6. Method Validation

The method validation was performed in accordance with the guidelines for bioanalytical method validation of the United States Food and Drug Administration (2018). The validation was evaluated in terms of specificity, precision, accuracy, linearity, recovery, matrix effect, stability, and dilution integrity in rat plasma.

#### 3.6.1. Specificity

Five different blank plasma samples were used to assess the ability of the method to detect and distinguish analytes from other materials present in the biological sample matrix. Interferences from endogenous compounds were assessed by comparing the blank plasmas with samples spiked with LLOQ concentrations of analyte or IS in the blank plasma. The detected responses that contribute to the interference component should be no more than 20% of the analyte response and 5% of the IS response at the LLOQ.

#### 3.6.2. Calibration Curve

The calibration curve was tested on 5 consecutive days and evaluated at 9 concentrations, except for the double blank and zero blank. The peak area ratios of the analytes and IS were plotted against the corresponding concentrations, and linear regression was applied. The curves were constructed by weighted 1/*x*^2^ regression. Each concentration value was required to be within ±20% for the LLOQ and ±15% for the remainder when back calculated against the full curve.

#### 3.6.3. LLOQ

The LLOQ was determined as the lowest concentration in the calibration range. This concentration was defined as having a signal-to-noise ratio of at least 5 as compared with the base noise of blank plasma and an accuracy (relative error, RE, %) between 80% and 120% of the theoretical value and a precision (relative standard deviation, RSD, %) not exceeding 20%.

#### 3.6.4. Precision and Accuracy

Intraday precision and accuracy were determined with five replicates at four concentrations across the linear range. Accuracy was determined by comparing the measured concentrations from the analysis with the nominal concentrations. The accuracy at each concentration level was assessed by relative error (RE, %). Precision was evaluated as the relative standard deviation (RSD, %), which is expressed as a percentage by dividing the standard deviation by the mean. The deviation was limited to within ±20% for the LLOQ, and the remainder of the QC samples were limited to within ± 15%.

#### 3.6.5. Recovery and Matrix Effect

The recovery and matrix effect were assessed using five different rat blank plasma samples. Recovery was assessed on three different QC samples (LQC, MQC, and HQC) by comparing QC samples prepared according to the above procedure and plasma samples spiked with analytes at the same concentrations after extraction. The matrix effect was assessed by comparing the analyte after extraction with a standard solution of the same concentration as that of the spiked plasma sample.

#### 3.6.6. Stability

Stability was evaluated with rat plasma at the four QC concentrations. Three freeze (−80 °C) and thaw (room temperature, 25 °C and stand for 2 h) cycles, long-term stability (−80 °C for 2 weeks), short-term stability (room temperature for 6 h), and post-extraction stability (12 h in the autosampler at 4 °C) were tested. The concentrations of the prepared stability samples were compared to the QC samples prepared on the day of analysis. The stability samples were considered stable if recovered within 15% compared to the QC sample concentration.

#### 3.6.7. Dilution Integrity

Dilution integrity was examined to allow for dilution without affecting the final concentration of biological samples above the upper limit of quantification. Samples with analyte concentrations 20 times higher than the upper limit of quantification were tested by diluting 50 and 100 times with blank plasma. Five replicates were performed, and the diluted samples were back calculated with the corresponding dilution factor to confirm the accuracy and precision.

### 3.7. Application to Pharmacokinetic Study in Rats

The rats were fasted for at least 12 h prior to the start of the experiments and freely supplied water. On the morning of the test, the rats were anaesthetized with a mixture of alfaxan and rompun (75:25, *v/v*). For oral administration studies, the carotid artery was cannulated using a polyethylene tube (PE 60, 0.76 mm i.d., 1.22 mm o.d., Becton Dickinson, Franklin Lakes, NJ, USA) for efficient blood sampling. For intravenous administration studies, the carotid artery and jugular veins were cannulated for blood sampling and drug administration, respectively. Each cannula was exteriorized to the dorsal side of the neck. Then, each rat was individually housed in a rat metabolism cage and allowed to move freely. A recovery time of 3–4 h from anesthesia was allowed before drug administration. The rats were randomly divided into six groups as follows: the first three groups were orally administered LDD-2614 (4 mL/kg dissolved in distilled water) using a gastric gavage tube at doses of 1, 5, and 20 mg/kg, and the remaining three groups were intravenously injected over 1 min with LDD-2614 (4 mL/kg dissolved in distilled water) at doses of 1, 5, and 20 mg/kg. Samples of approximately 0.2 mL of blood were immediately collected via the carotid artery at 0 (before administration), 5, 15, 30, 60, 90, 120, 180, 240, 360, 480, and 1440 min after oral and intravenous administration. For intravenous administration, a sampling time of 1 min after administration was also added, and the remaining times were the same. The blood samples were immediately centrifuged at 12,000 rpm for 5 min. Each plasma sample was transferred into a new microtube and stored at −80 °C until used for LC–MS/MS analysis.

### 3.8. Data Analysis

All data are expressed as mean ± standard deviation. The pharmacokinetic parameters were calculated using Phoenix WinNonlin 2.1 (Pharmasight, Mountain View, CA, USA). A noncompartmental model was used.

## 4. Conclusions

In this study, a rapid, selective, and sensitive LC-MS/MS method for quantifying LDD-2614 in rat plasma was developed and validated for the first time. Plasma extraction with ethyl acetate provided analyte reproducibility and good recovery. This method has a short analysis time; suitable linearity, accuracy, and precision; and no matrix effect. The analytes were also stable under all conditions tested in the matrix. This method was successfully applied to biological samples obtained after oral and intravenous administration of LDD-2614 to rats, allowing the concentrations to be measured. Therefore, this new bioanalytical method has been proven to be suitable for pharmacokinetic studies. This study provides useful information for further research on the pharmacokinetics of LDD-2614, a newly discovered FLT3 inhibitor.

## Figures and Tables

**Table 1:** Optimized mass spectrometer parameters and multiple reaction monitoring (MRM) transitions of the LDD-2614 and LDD-2633 (IS).

Compounds	MRM Transition (*m/z*)	DP ^1^	EP ^2^	CEP ^3^	CE ^4^	CXP ^5^
LDD-2614	426.2→113.1	51	8.5	20	29	4
IS	390.2→113.1	41	11	24	27	4

^1^ DP, declustering potential; ^2^ EP, entrance potential; ^3^ CEP, collision cell entrance potential; ^4^ CE, collision energy; ^5^ CXP, collision cell exit potential.

**Table 2:** Matrix-matched calibration curve parameters for LDD-2614 (*n* = 5).

No.	Typical Equation	*r* ^2^
1	*y* = 0.01079*x* − 0.0003576	0.9991
2	*y* = 0.01095*x* − 0.0001499	0.9996
3	*y* = 0.01088*x* − 0.0002181	0.9992
4	*y* = 0.01101*x* − 0.0002802	0.9995
5	*y* = 0.01004*x* − 0.0001232	0.9997

**Table 3:** Accuracy and precision of calibration curve of LDD-2614 (*n* = 5).

Nominal Concentration (ng/mL)	Measured Concentration (ng/mL) ^1^	Precision (RSD,%)	Accuracy (RE,%)
0.1	0.10 ± 0.01	5.5	1.5
0.5	0.50 ± 0.02	3.6	0.9
1	0.97 ± 0.04	4.4	−3.0
5	4.97 ± 0.13	2.6	−0.5
10	9.88 ± 0.36	3.6	−1.2
50	49.0 ± 1.15	2.3	−2.1
100	99.4 ± 4.33	4.4	−0.6
200	192.6 ± 6.30	3.3	−3.7
500	479.7 ± 4.65	1.0	−4.1

^1^ Each value is expressed as mean ± standard deviation.

**Table 4:** Intraday and interday accuracy and precision of LDD-2614 (*n* = 5).

Nominal Concentration (ng/mL)	Intra-day			Inter-day		
	Measured Concentration (ng/mL) ^1^	Precision (RSD,%)	Accuracy (RE,%)	Measured Concentration (ng/mL) ^1^	Precision (RSD,%)	Accuracy (RE,%)
0.1	0.11 ± 0.01	7.1	7.0	0.11 ± 0.01	9.0	8.7
0.3	0.32 ± 0.01	3.1	7.1	0.30 ± 0.02	7.5	1.1
150	145.2 ± 4.40	3.0	−3.2	147.5 ± 4.53	3.1	−1.7
400	396.7 ± 12.1	3.0	−0.8	393.4 ± 12.0	3.1	−1.7

^1^ Each value is expressed as mean ± standard deviation.

**Table 5:** Recovery and matrix effect of LDD-2614 in rat plasma (*n* = 5). Each value is expressed as mean ± standard deviation.

Compounds	Nominal Concentration (ng/mL)	Recovery		Matrix Effect	
		Recovery (%)	RSD (%)	Matrix Effect (%)	RSD (%)
LDD-2614	0.3	103.9	5.3	100.6	4.3
	150	100.6	1.7	101.0	1.4
	400	98.9	2.4	99.5	2.2
IS	1000	98.0	6.1	98.1	5.3

**Table 6:** Stability of LDD-2614 in rat plasma (*n* = 5).

	Nominal Concentration (ng/mL)	Measured Concentration (ng/mL) ^1^	Precision (RSD,%)	Accuracy (RE,%)
Three Freeze-thaw (3 cycles)	0.3	0.33 ± 0.02	5.8	8.6
	150	149.1 ± 1.31	0.9	−0.6
	400	398.9 ± 6.04	1.5	−0.3
Long-term stability (−80 °C, 2 weeks)	0.3	0.29 ± 0.02	6.1	−4.8
	150	146.1 ± 0.65	0.4	−2.6
	400	378.1 ± 17.7	4.7	–5.5
Short-term stability (room temp, 6 h)	0.3	0.30 ± 0.03	10.8	−0.1
	150	144.6 ± 6.46	4.5	−3.6
	400	395.1 ± 13.0	3.3	−1.2
Autosampler stability (4 °C, 12 h)	0.3	0.30 ± 0.03	8.5	−1.1
	150	147.8 ± 2.98	2.0	−1.5
	400	410.2 ± 24.3	6.0	1.5

^1^ Each value is expressed as mean ± standard deviation.

**Table 7:** Pharmacokinetics parameter of LDD-2614 after intravenous and oral administration. Each value is expressed as mean ± standard deviation.

Parameter	1 mg/kg	5 mg/kg	20 mg/kg
Intravenous	(*n* = 7)	(*n* = 5)	(*n* = 6)
AUC_last_ (μg·min/mL)	38.1 ± 18.5	210.5 ± 64.7	864.5 ± 149.9
AUC/Dose	38.1 ± 18.5	42.1 ± 12.9	43.2 ± 7.5
CL (mL/min/kg)	30.7 ± 13.6	20.8 ± 8.4	23.2 ± 3.7
MRT (min)	272.0 ± 32.8	285.5 ± 31.1	352.9 ± 41.6
T_1/2_ (min)	294.5 ± 32.5	198.9 ± 34.5	202.5 ± 46.7
V_d,ss_ (L/kg)	9.62 ± 3.38	6.42 ± 2.44	8.67 ± 2.08
Oral	(*n* = 6)	(*n* = 6)	(*n* = 5)
AUC_last_ (μg·min/mL)	4.43 ± 0.39	23.4 ± 15.2	61.1 ± 24.8
AUC/Dose	4.43 ± 0.39	4.69 ± 3.05	3.05 ± 1.24
C_max_ (ng/mL)	5.96 ± 1.77	43.7 ± 8.2	119.6 ± 33.4
T_max_ (min) ^1^	480 (480–480)	480 (360–480)	360 (240–480)
F (%)	11.7	11.1	7.1

^1^ Each value is expressed as median with ranges (parenthesis). AUC_last_, area under plasma concentration-time curve from zero to last time; CL, the time averaged total body clearance; MRT, mean residence time; T_1/2_, terminal half-life; V_d,ss_, apparent volume of distribution at steady state; C_max_, maximum plasma concentration; T_max_, time to reach a C_max_; F, bioavailability.
